# expHRD: an individualized, transcriptome-based prediction model for homologous recombination deficiency assessment in cancer

**DOI:** 10.1186/s12859-024-05854-y

**Published:** 2024-07-12

**Authors:** Jae Jun Lee, Hyun Ju Kang, Donghyo Kim, Si On Lim, Stephanie S. Kim, Gahyun Kim, Sanguk Kim, Jin-Ku Lee, Jinho Kim

**Affiliations:** 1 Computational Cancer Genomics Groups, Spanish Cancer Research Center (CNIO), Madrid, Spain; 2 Genomic Medicine Institute, Medical Research Center, Seoul National University College of Medicine (SNUCM), Seoul, 03080 Republic of Korea; 3 Department of Biomedical Sciences, Seoul National University College of Medicine (SNUCM), Seoul, 03080 Republic of Korea; 4 Department of Anatomy and Cell Biology, Seoul National University College of Medicine (SNUCM), Seoul, 03080 Republic of Korea; 5 Pohang University of Science and Technology (POSTECH), Pohang, Gyeongbuk 37673 Republic of Korea; 6 Precision Medicine Center, Future Innovation Research Division, Seoul National University Bundang Hospital (SNUBH), Seongnam, Gyeonggi-do 13620 Republic of Korea; 7 Department of Genomic Medicine, Seoul National University Bundang Hospital, Seongnam, Gyeonggi-do 13620 Republic of Korea; 8 Department of Laboratory Medicine, Seoul National University Bundang Hospital, Seoul National University College of Medicine, Seongnam, Gyeonggi-do 13620 Republic of Korea

**Keywords:** Homologous recombination deficiency, scarHRD, Transcriptome, Elastic net regression, Bootstrap

## Abstract

**Background:**

Homologous recombination deficiency (HRD) stands as a clinical indicator for discerning responsive outcomes to platinum-based chemotherapy and poly ADP-ribose polymerase (PARP) inhibitors. One of the conventional approaches to HRD prognostication has generally centered on identifying deleterious mutations within the *BRCA1/2* genes, along with quantifying the genomic scars, such as Genomic Instability Score (GIS) estimation with scarHRD. However, the scarHRD method has limitations in scenarios involving tumors bereft of corresponding germline data. Although several RNA-seq-based HRD prediction algorithms have been developed, they mainly support cohort-wise classification, thereby yielding HRD status without furnishing an analogous quantitative metric akin to scarHRD. This study introduces the expHRD method, which operates as a novel transcriptome-based framework tailored to n-of-1-style HRD scoring.

**Results:**

The prediction model has been established using the elastic net regression method in the Cancer Genome Atlas (TCGA) pan-cancer training set. The bootstrap technique derived the HRD geneset for applying the expHRD calculation. The expHRD demonstrated a notable correlation with scarHRD and superior performance in predicting HRD-high samples. We also performed intra- and extra-cohort evaluations for clinical feasibility in the TCGA-OV and the Genomic Data Commons (GDC) ovarian cancer cohort, respectively. The innovative web service designed for ease of use is poised to extend the realms of HRD prediction across diverse malignancies, with ovarian cancer standing as an emblematic example.

**Conclusions:**

Our novel approach leverages the transcriptome data, enabling the prediction of HRD status with remarkable precision. This innovative method addresses the challenges associated with limited available data, opening new avenues for utilizing transcriptomics to inform clinical decisions.

**Supplementary Information:**

The online version contains supplementary material available at 10.1186/s12859-024-05854-y.

## Background

The effective repair of DNA damage has been a crucial evolutionary mechanism for sustaining the DNA integrity of mammalian cells, given their constant exposure to a spectrum of endogenous and exogenous DNA-damaging events such as irradiation, free radicals, and mutagenic chemicals [1, 2]. A prominent form of DNA damage is the DNA double-strand break (DSB), which is fatal to cell survival if left unrepaired [3]. Consequently, eukaryotic cells have developed an intricate DNA–DSB repair machinery characterised by two major mechanisms: homologous recombination (HR) and non-homologous end joining (NHEJ) [4].

While NHEJ directly fuses damaged DNA segments without a template, HR synthesises and recovers the lost DNA segments using a homologous chromosome as a blueprint [4]. Therefore, NHEJ primarily recovers numerous DSBs quickly, albeit with a higher error probability compared with that of HR. Cells with compromised HR frequently resort to inaccurate and error-prone DNA–DSB repair mechanisms, imperilling genomic integrity [5]. Core molecules contributing to the HR pathway include BRCA1/2, ATM/ATR, RAD51, EMSY, and PTEN [6]. Dysfunctional HR-related molecules frequently engender HR deficiency (HRD) [6, 7], which holds profound implications across various malignancies, including ovarian, triple-negative breast, prostate, and pancreatic cancers [8–10]. More importantly, HRD has earned status as a clinically verified companion diagnostic (CDx) marker predicting therapeutic response to various anti-cancer agents, including platinum-based chemotherapies and poly ADP-ribose polymerase (PARP) inhibitors [11, 12]. Noteworthy among these inhibitors are olaparib, niraparib, and rucaparib, sanctioned for treating HRD-associated malignancies and demonstrating striking survival benefits, particularly in ovarian and triple-negative breast cancers with HRD [13–18].

Consequently, robust identification of HRD in cancer has become critical for tailoring precision therapy strategies, especially in the context of PARP inhibition [11]. The predominant method for HRD detection entails identifying deleterious mutations in HR-related genes, mainly *BRCA1* or *2* [19]. However, this method’s scope is limited, as *BRCA1/2* mutations encompass less than half of HRD cases [20]. The detection of “genomic scars,” indicative of loss of heterozygosity (LOH), large-scale state transition (LST), and telomeric allelic imbalance (TAI), provides a long-term manifestation of significant structural variations in HRD [21–23]. Myriad’s Mychoice CDx (MC-CDx) amalgamates *BRCA1/2* mutations and genomic instability score (GIS), the sum of LOH, LST, and TAI, as a response indicator to PARP inhibitors and a prognostic factor in breast and ovarian cancer [22, 24]. Yet, even this method has limitations, as the GIS calculation algorithm basically relies on normalisation using corresponding matched germline data. In the clinic, normalisation using pooled germline data is applied in GIS calculation because germline DNA copy number data are frequently missing, which may lead to difficulty in diagnosing HRD status in particular cases.

Alternatively, transcriptomic profiling of HR pathways has revealed specific gene signatures significantly correlated with HRD status. For instance, Peng et al*.* identified 230 HR-associated genes by analysing differentially expressed genes between cells of WT and those with simultaneous knockdowns of *BRCA1, RAD51,* and *BRIT1* genes in MCF-10A cells [25]. Recent attempts at HRD-RNA algorithm development have adopted logistic regression models for the prediction of HR-related genetic variants in ovarian and breast cancers using cancer transcriptome data [26]. Furthermore, the detection of aberrant transcripts has been suggested as a valuable diagnostic tool for identifying HRD tumours [27]. However, most existing methods for analysing HRD-specific genes rely on cohort-wise and clustering-based classifications, which pose limitations in clinic application as genetic diagnoses frequently require individualised assessment.

In this study, we developed a machine-learning-based algorithm that reliably correlates with the scarHRD score through RNA-seq analysis of designated samples. Leveraging the pan-cancer cohort from the Cancer Genome Atlas (TCGA), we devised and validated this HRD prediction performance. A total of 356 genes were selected for genomic scar prediction by the elastic net regression and consecutive bootstrap methods. To enable n-of-1-style HRD predictions, we further developed the expHRD algorithm by adopting single-sample geneset enrichment analysis (ssGSEA) methods, which extracts cohort-independent HRD predictions in the cancer transcriptome. We evaluated the clinical feasibility of expHRD methods in TCGA-OV test sets and the Genomic Data Commons (GDC) ovarian cancer cohort [28]. Moreover, we developed a web server demonstrating the expHRD and predicted HRD scores by easily uploading the RNA-seq data of the users’ interests.

## Methods

### Data collection and GIS calculation

#### TCGA and PanCanAtlas data collection

A comprehensive dataset comprising 10,068 tumours across 35 distinct cancer types was assembled from TCGA pan-cancer cohort. SNP array data, processed through allele-specific Copy Number Analysis of Tumour Samples (ASCAT2), was retrieved from the Genomic Data Commons Data Portal (https://portal.gdc.cancer.gov). Additionally, whole transcriptomic sequencing (WTS) data, providing gene-level transcription estimates in RSEM-normalised counts, were obtained from Firebrowse (http://firebrowse.org/).

For validation purposes, an exclusive set of GDC TCGA-OV samples (n = 112) was incorporated, distinct from the TCGA-OV samples (ovarian cancer; n = 287). WTS/SNP array data and survival information were sourced from Xenabrowser (https://xenabrowser.net/) and the GDC Data Portal (https://portal.gdc.cancer.gov), respectively [29].

#### scarHRD calculation

Genomic instability, represented by scarHRD, was computed using the R package “scarHRD”, based on copy number segments derived from SNP array data [30]. The default parameters of the R package were utilised [23]. ScarHRD encompasses three subtypes of genomic instability: LOH [31], LST [32], and TAI [33]. Specifically, LOH denotes the loss of a chromosomal region exceeding 15 Mb or entire chromosome depletion. LST is characterised by chromosome breaks, resulting in two separated regions exceeding 10 Mb with a distance less than 3 Mb. TAI represents regions with allelic imbalances extended to the telomeric region.

### Development of an HRD prediction model

#### Processing of RNA-sequencing data

For the training and test sets, gene expression data were sourced from FIREBROWSE across various cancer cohorts, utilizing RSEM-rawcount values. Normalization was performed using DESEQ2 (version 1.34.0). Prior to normalization, we excluded genes lacking HGNC symbols and also filtered out lowly expressed genes as part of DESEQ2’s normalization process. Furthermore, genes exhibiting zero expression levels were removed during the preliminary filtering stage. In case of TCGA samples, 20,052 gene were composed of RNA-sequencing data which was already processed by the pipeline of RSEM-normalized counts, obtained from Firebrowse (http://firebrowse.org/). Additionally, the number of protein coding genes in GDC TCGA-OV samples were processed by the same pipelines of TCGA samples. AOCS samples were obtained from Gene Expression Omnibus (GSE209964), which was already processed by the Garsed et al. [34]. Sample annotation with batch process in AOCS consists of three parts (1: AOCS sample (n = 75), 2: MAOC sample (n = 30), and MMAC sample (n = 26)). The ‘Deseq2’ (ver 1.34.0) R package [34] facilitated the acquisition of normalized gene-expression data by VST normalization for test, validation, and AOCS sets. Additional batch effect remove in AOCS samples was done by ‘limma’ R packages [35].

#### Establishment of a machine-learning model

Construction: Filtering based on differentially expressed gene (DEG) analysis yielded a set of 4436 genes associated with the scarHRD score’s continuous distribution. The machine-learning model aimed to predict the HRD score (target values; Y) through gene-expression values (training data; X), employing regression algorithms. The efficacy of various methods—Ridge, Lasso, elastic net regression, Support Vector Machine, Gradient Boosting Model, and Multilayer Perceptron—was initially compared (Table S1).

-Ridge: A linear regression model incorporating L2 regularisation through a penalty term. The hyperparameter alpha (α) regulates the model’s regularisation strength.

$$\underset{w}{min}||Xw-y|{|}_{2}^{2}+\alpha ||w|{|}_{2}^{2}$$

Lasso: This estimation model entails L1 regularisation, effectively reducing the number of features. The hyperparameter encompasses the number of iterations needed to counteract overfitting or underfitting issues.

$$\underset{w}{min}\frac{1}{2{n}_{\text{samples}}}||Xw-y|{|}_{2}^{2}+\alpha ||w|{|}_{1}$$

Elastic net regression: A composite of Lasso and Ridge, encompassing both L1 and L2 regularisation. Hyperparameters encompass L1 and L2 penalties and the number of iterations.

$$\underset{w}{min}\frac{1}{2{n}_{\text{samples}}}||Xw-y|{|}_{2}^{2}+\alpha \rho ||w|{|}_{1}+\frac{\alpha (1-\rho )}{2}||w|{|}_{2}^{2}$$

Support Vector Machine (SVM): A supervised learning method for regression, SVM classifies samples by different functions using linear or radial basis functions. Hyperparameters include the regularisation parameter for both linear and RBF models.

Gradient Boosting Regressor: An ensemble method necessitating the learning rate as a hyperparameter. It involves repeated training steps to mitigate overfitting or underfitting, with significant features identified by the model.

Multilayer Perceptron: Utilising multiple layers, this model predicts target values, with layer count and constraint function serving as configurational parameters.

### Performance evaluation of machine-learning models

To comprehensively assess the efficacy of each machine-learning model, a range of classification metrics were employed, encompassing accuracy, specificity, sensitivity, precision, F-score, AUC-ROC, AUC-PR, and Matthews Correlation Coefficient (MCC) score. Distinct from conventional classification algorithms, we transformed the outcome of regression into a binary classification, distinguishing between positive and negative status (positive: scarHRD score ≥ 42, negative: scarHRD < 42). Subsequently, model performance was evaluated through a comparison involving confusion matrices.

$$Accuracy= \frac{TP+TN}{TP+FP+TN+FN}$$

$$Sensitivity= \frac{TP}{TP+FN}$$

$$Specificity= \frac{TN}{TN+FP}$$

$$Precision = \frac{TP}{TP+FP}$$

$$F1 \; score= \frac{2TP}{2TP+FP+FN}$$

$$MCC= \frac{TP*TN-FP*FN}{\sqrt{\left(TP+FP\right)*\left(TP+FN\right)*\left(TN+FP\right)*(TN+FN)}}$$

The area under the ROC curve (AUC-ROC) and area under precision curves (AUC-PR) were computed based on the sensitivity and (1−Specificity) curves, respectively.

The Lasso, Ridge, and Elascticnet models were initially trained using “GridSearchCV” from the Python library “Scikit-learn” with five-fold cross validation, obtaining optimal hyperparameters. Specifically, we tested the effect of the cross-validation process in Elastincnet from three-fold to seven-fold training to get the best performance of training with an R-squared score and the optimal number of selected gene sets, the second gene sets. All machine-learning training, cross validation, and performance comparison were performed using the Python library ‘Scikit-learn’ for the construction of models and the R package ‘MultipleROC’ for the AUC calculation, respectively (https://jmlr.org/papers/v12/pedregosa11a.html).

### Machine-learning prediction in the TCGA-*pan cancer* cohort

For test set evaluation, 20% of samples were selected (n = 2027). Pearson’s correlation between scarHRD and predicted HRD was calculated within the test set, and the two-sided t-test determined the *P* value. The mean square error (MSE) was calculated using scarHRD and predicted HRD values, yielding the following equations:

$${\text{MSE}}(y,\hat{y})=\frac{1}{{n}_{\text{samples}}}\sum_{i=0}^{{n}_{\text{samples}}-1}({y}_{i}-{\hat{y}}_{i}{)}^{2}$$

The root mean squared error (RMSE) was subsequently calculated using the Python library ‘Scikit-learn’.

### Bootstrap process and expHRD calculation

#### Bootstrap process

To obtain robust HRD-related gene sets, the bootstrap procedure hinged upon elastic net regression model outcomes, with an initial feature count of 2538 and relevant hyperparameters. The distribution of feature weights was based on the initial elastic net model. Initially, training samples were randomly chosen for 100 iterations, with duplication disregarded. Subsequently, the model was retrained with identical specifications, leading to feature weight determination. Features with non-zero values across at least 98 instances in 100 repetitions were selected.

#### Calculation of expHRD

Rooted in the single-sample gene set analysis (ssGSEA) method, expHRD computation entailed assessing the gene-expression pattern of individual samples. Gene sets derived from the bootstrap procedure constituted the basis for ssGSEA calculations. HRD-positive and HRD-negative gene sets contributed to the calculation using the following equation:

$$expHRD=ssGSEA \; score \; in \; HRD \; positive-ssGSEA \; score \; in \; HRD \; neagative$$

Each ssGSEA score was calculated from the R package ‘GSVA’ [36] with the function ‘ssGSEA’ and our selected positive and negative gene sets.

### Survival analysis

Survival status data were sourced from the GDC Data Portal, aligned with the latest follow-up version for each cancer type. The analysis involved two groups, categorised based on scarHRD and expHRD. For this analysis, only the TCGA-OV and GDC TCGA-OV cohorts were considered. The scarHRD group criterion was established at a specific scarHRD score of 42 [37]. ExpHRD score criteria were determined by the best AUC score prediction outcome, employing the ‘scipy’ Python library for Pearson’s correlation and the ‘MultipleROC’ R package. Median survival, defined as the time at which survival probability reached 50%, was determined using Kaplan–Meier estimation. The log-rank test facilitated *P* value calculation for group comparison, and survival probability was reported with 95% confidence intervals.

## Results

### Development of the HRD prediction model using the transcriptome

**Fig. 1:**
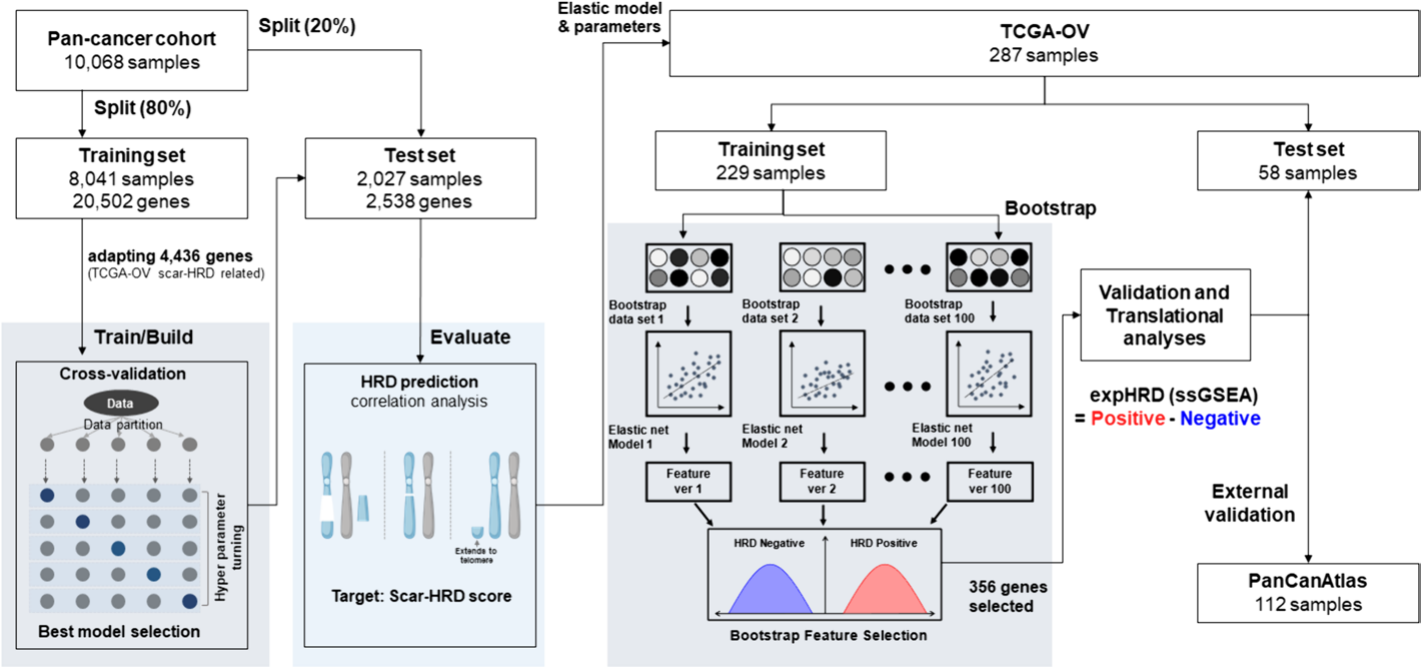
Schematic representation of transcriptome-based HRD prediction model development and validation. Schematic illustration describing the overall process of serial machine-learning training and validation for the development of an HRD prediction algorithm in the cancer transcriptome

A comprehensive dataset of 10,068 samples representing 34 cancer types from the TCGA-pan cancer cohort, encompassing both tumour and blood samples with SNP array copy number variation and WTS, was harnessed for training, development, and evaluation of the HRD prediction model (Fig. 1). The scarHRD score for each sample was derived by aggregating HRD-associated genomic scars—namely, LOH, LST, and TAI.

In a sequential manner, the dataset was stratified into two subsets: a training set (n = 8041) comprising 80% of the samples, and a test/validation set (n = 2027) comprising the remaining 20%. The training set included 1422 HRD and 6619 HR-proficient (HRP) samples, while the test/validation set contained 353 HRD and 1674 HRP samples. Gene selection was executed by retaining 20,502 genes from RNA-seq data and filtering down to 4436 DEG based on scarHRD status within the TCGA-OV set—an essential component of the clinical HRD prediction algorithm evaluation.

Gene feature optimisation was accomplished through iterative machine-learning training employing elastic net and bootstrap methodologies. A total of 356 genes were meticulously chosen to facilitate the calculation of expHRD, which has applicability at the individual patient level in clinical contexts. Subsequent validation processes encompassed internal validation (TCGA-OC test, n = 58) and external validation (GDC PanCanAltas, ovarian cancer, n = 112).

### Performance evaluation of the HRD prediction model

**Fig. 2:**
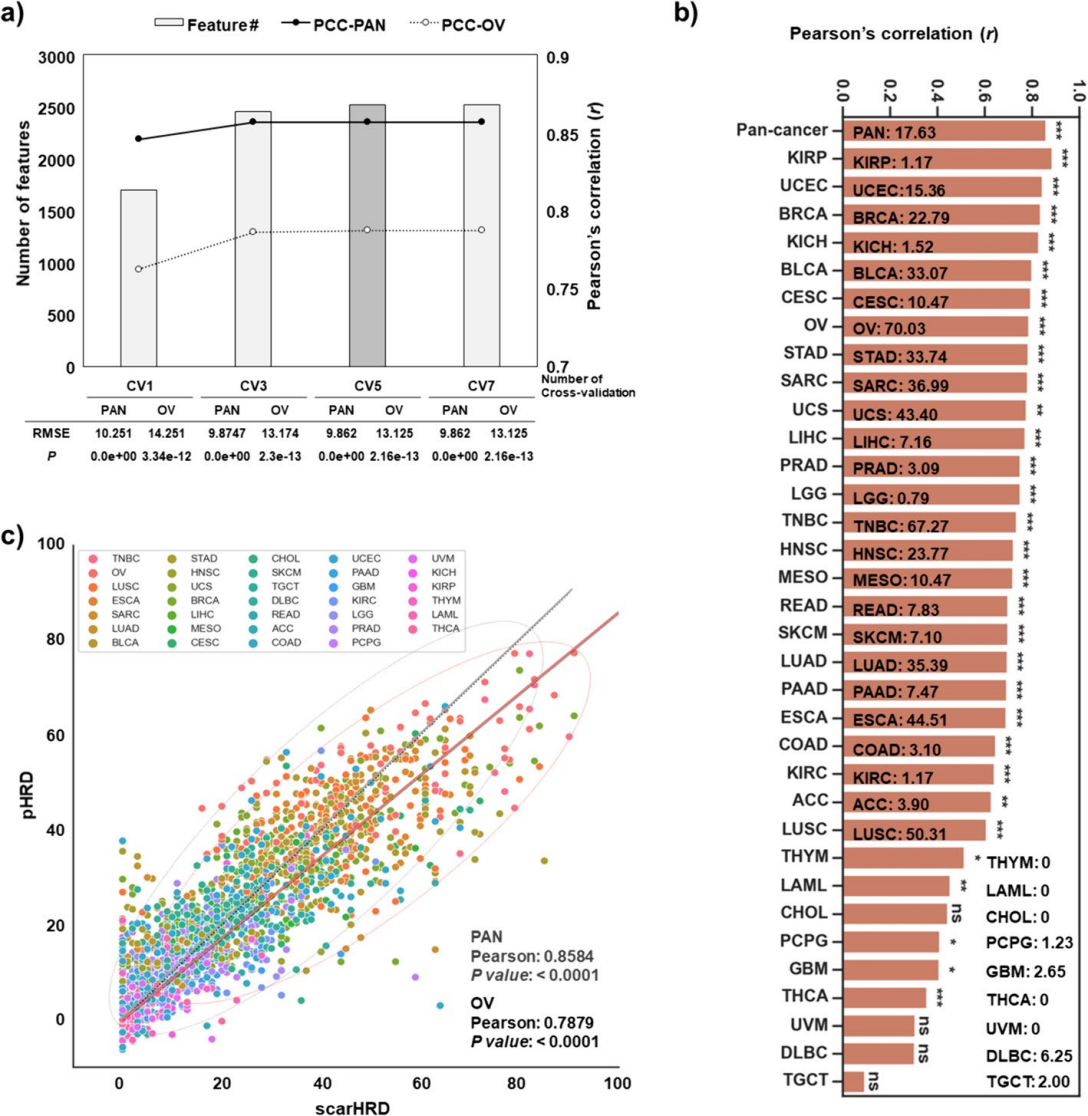
Evaluation of the HRD prediction model in the TCGA-pan cancer. **a** Cross-validation analysis of the machine-learning model. The x-axis denotes the number of elastic net cross-validation iterations. The left-y-axis signifies the count of features (genes), while the right-y axis indicates Pearson’s correlation coefficient (PCC) with the scarHRD score post-machine learning. Black closed circles linked by solid lines and white circles connected by dotted lines correspond to the gene count and PCC, respectively, across each cross-validation step. **b** Correlation pattern across TCGA-pan cancer cohorts. Bar graph depicting the PCC between the predicted HRD score and scarHRD score in the TCGA-pan cancer test set, encompassing various cancer types including KIRP (kidney renal clear papillary cell carcinoma), UCEC (uterine corpus endometrial carcinoma), BRCA (breast invasive carcinoma), KICH (kidney chromophobe), BLCA (bladder urothelial carcinoma), CESC (cervical squamous cell carcinoma and endocervical adenocarcinoma), OV (ovarian serous cystadenocarcinoma), STAD (stomach adenocarcinoma), SARC (sarcoma), UCS (uterine carcinosarcoma), LIHC (liver hepatocellular carcinoma), PRAD (prostate adenocarcinoma), LGG (brain lower grade glioma), TNBC (triplet negative breast cancer), HNSC (head and neck squamous cell carcinoma), MESO (mesothelioma), READ (rectum adenocarcinoma), SKCM (skin cutaneous melanoma), LUAD (lung adenocarcinoma), PAAD (pancreatic adenocarcinoma), ESCA (esophageal carcinoma), COAD (colon adenocarcinoma), KIRC (kidney renal clear cell carcinoma), ACC (adrenocortical carcinoma), LUSC (lung squamous cell carcinoma), THYM (thymoma), CHOL (cholangiocarcinoma), PCPG (pheochromocytoma and paraganglioma), GBM (glioblastoma multiforme), THCA (thyroid carcinoma), UVM (uveal melanoma), DLBC (lymphoid neoplasm diffuse large B-cell lymphoma), and TGCT (testicular germ cell tumours). Significance levels denoted as *, **, and *** indicate *P*-values < 0.05, < 0.001, and < 0.0001, respectively. The frequency of HRD (scarHRD score ≥ 42) in each tumour type is displayed. **c** Correlation between scarHRD and predicted HRD score (pHRD) in the TCGA-pan cancer test set. Pearson’s correlation-regression line was calculated, with the dark dotted line illustrating pan-cancer correlation and the red line representing TCGA-OV set correlation. The numeric number in each bar plot represents the frequency of HRD positive samples in cancer types. Frequency: the number of HRD positive sample / the number of sample with scarHRD score

The expression data of initially selected genes (n = 4436) from the pan-cancer training set were subjected to various linear regression models, such as Ridge, Lasso, elastic net regression, SVM, Gradient Boosting Regressor (GBM), and Multilayer Perceptron (MLP), for the prediction of corresponding sample scarHRD scores (Table S1). The gene count ranged from 862 to 4436, yielding R-squared values varying from 0.6375 to 0.7364, dependent upon the regression models (Table 1). The R-squared value is indicative of the predictive accuracy of our machine learning model, representing the proportion of variance in the actual scarHRD scores that our model can explain. Notably, the elastic net regression model, among the regression options, exhibited the highest correlation with the scarHRD score and was subsequently chosen for refining the prediction algorithm. Five-fold cross-validation demonstrated feature number saturation (n = 2538) and yielded a Pearson’s correlation coefficient (PCC) of 0.858 (*P* < 0.0001) and 0.788 (*P* < 0.0001) in the TCGA-pan cancer and OV test cohorts, respectively (Fig. 2a). The Pearson’s correlation coefficient measures the linear correlation between the actual scarHRD scores and the HRD scores predicted by our machine learning model, providing insight into the strength and direction of this linear relationship.

**Table 1:** Performance evaluation of various machine-learning methods

	Elastic net		Lasso		Ridge		GBM		SVR (Rbf)		SVR (linear)		MLP	
Cancer	34 (33 + TNBC)													
Input genes (n)	4436 (result of DEG of OV)													
Selected gens (n)	2538		862		4436		2755		x		4436		x	
	R2	RMSE	R2	RMSE	R2	RMSE	R2	RMSE	R2	RMSE	R2	RMSE	R2	RMSE
PAN	0.7364	9.8624	0.7319	9.9462	0.6375	11.566	0.6943	10.622	0.7238	10.097	0.7163	10.232	0.7129	10.294
OV	0.592	13.125	0.59	13.158	0.53	14.088	0.5408	13.925	0.5568	13.68	0.5235	14.184	0.6243	12.594
TMBC	0.4682	17.765	0.4576	17.941	0.5489	16.361	0.3074	20.273	0.4417	18.201	0.5492	16.355	0.6245	14.927

Following elastic net regression, the R-squared values were as follows: pan-cancer, 0.7364; ovarian cancer (OV), 0.592; and triple-negative breast cancer (TNBC), 0.4682. Similar conclusions were drawn from the performance metrics of various machine-learning models for predicting HRD-high samples (scarHRD ≥ 42) within the TCGA-OV test set (Table S2).

The PCC of the HRD prediction score with scarHRD was 0.8584 (*P* < 0.0001) in the pan-cancer test set. In most cancer types, excluding those with limited clinical significance in relation to HRD status (e.g., cholangiocarcinoma, uveal melanoma, diffuse large B-cell lymphoma, testicular germ cell tumour), a significant correlation with the scarHRD score was observed (Fig. 2b, c). Notably, HRD prediction value in clinically relevant cancer types—such as OV, breast cancer (BRCA), TNBC, and prostate adenocarcinoma (PRAD)—displayed substantial correlations with scarHRD score (PCC > 0.7). Particularly in the case of TCGA-OV, a validation cohort with clinical relevance, the HRD prediction score showcased a significant and remarkable correlation with the scarHRD score (PCC = 0.7879, *P* = 2.1556e−13, Fig. 2c).

### Bootstrap for feature optimisation of the HRD prediction model

Further refinement of gene features was achieved through sequential machine-learning analysis employing elastic net and bootstrap techniques (Fig. 1). The gene feature count for HRD score prediction was optimised, reducing from 4436 to 2538 via elastic net regression (Ela). Subsequently, a second-round elastic net regression (Re-Ela) further streamlined this number to 2337. To achieve maximal gene feature optimisation, we embraced the bootstrap method, maintaining elastic net regression parameters. Through this iterative process involving random sampling and training set generation (n = 229) over 100 iterations, 356 genes consistently emerged in 98 out of 100 bootstrap instances (Fig. S1a). Notably, the PCC with scarHRD score improved from 0.533 to 0.767 across optimisation stages, while gene features dwindled from 4436 to 356.

The final selection encompassed 356 gene features classified as positively correlated (n = 183) and negatively correlated (n = 173), out of which a significant majority—94.8% for positively correlated and 94.0% for negatively correlated—were identified as coding sequences (CDS) (Fig. S1b and S2). In addition, we used the EnrichR package to perform gene set enrichment analysis to investigate the association of HRD score with positively associated genes (Table S3) and negatively associated genes (Table S4) with specific pathways. The results revealed that genes such as *ATR* and *AURKA* and pathways related to “Regulation of DNA repair” and “Cell cycle process” exhibited significant positive correlation with HRD score. On the other hand, genes and pathways associated with “Cell cycle” or “DNA damage checkpoint”, including *BRCA1*, demonstrated a negative correlation with HRD.

### Development and validation of the expHRD algorithm

**Fig. 3:**
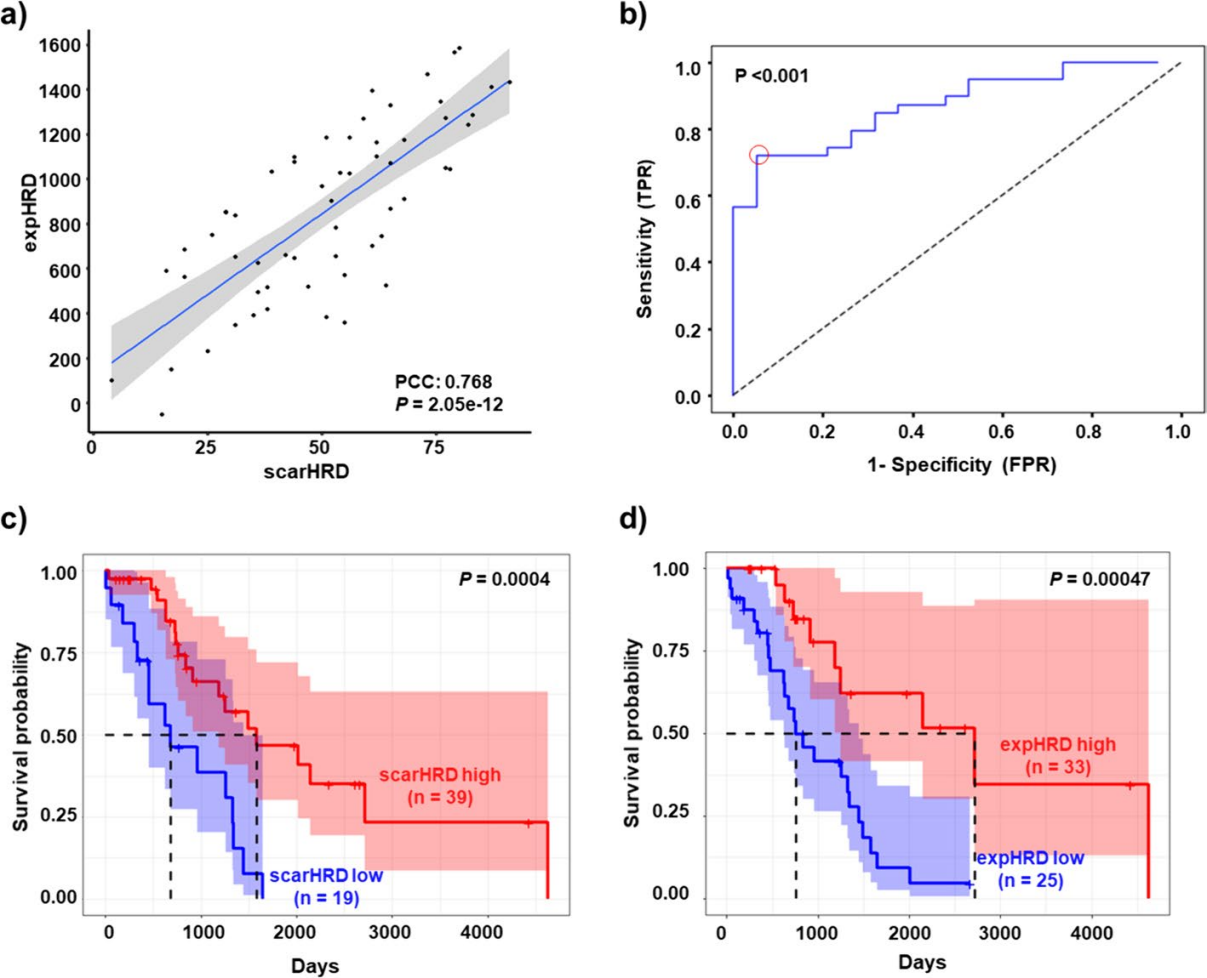
Validation of expHRD performance in the TCGA-OV test set. **a** Pearson’s correlation between expHRD and scarHRD in the TCGA-OV test set (n = 58, PCC = 0.768, *p* = 2.045e−12). The blue line denotes the regression line, while the shaded area represents the 95% confidence interval (CI). **b** Receiver operating characteristic (ROC) curve plotting sensitivity against 1-specificity values for expHRD score’s capacity to predict scarHRD-high instances within TCGA-OV test samples. **c**, **d** Kaplan–Meier overall survival analysis contrasting patients with high vs. low scarHRD (**c**) or expHRD (**d**) status within the TCGA-OV test set. *P*-values obtained via the log-rank test

Calculation of expHRD was accomplished through single-sample gene set analysis (ssGSEA) using a gene set derived from the bootstrap process. This computation was executed using a newly devised equation to tailor the expHRD values per sample: The expHRD score is determined by subtracting the ssGSEA score of HR-negative genes from that of HR-positive genes. Notably, the PCC of expHRD against scarHRD score was 0.768 (*P* = 2.045e−12) in the TCGA-OV test set (Fig. 3a) and 0.633 (*P* = 6.655e−14) in the GDC ovarian cancer cohort (Fig. S3a). For evaluating the predictive performance of expHRD concerning scarHRD-high samples, receiver operating characteristics (ROC) curve analysis was conducted, revealing area under curve (AUC) values of 0.872 and 0.806 in TCGA-OV (Fig. 3b) and the GDC ovarian cancer cohort (Fig. S3b), respectively. To classify samples as having high or low expHRD, we utilized a regression analysis to align expHRD scores with those obtained from scarHRD, thereby standardizing our scoring against a recognized metric. Samples exhibiting an expHRD score of 1000 or higher were classified as having a high level of HRD.

Next, we analyzed the association between the mutation and promoter methylation of *BRCA1/2* genes, and the distribution of expHRD calculated using our algorithm in the TCGA-OV and -TNBC test set (n = 58 and 12, respectively). The results revealed a statistically significant enrichment of *BRCA1/2* mutations and methylation in samples with higher expHRD scores (expHRD ≥ 1000; *P* = 4.973e−04; Fig. S4). The accuracy and precision for predicting scarHRD were 0.71 and 0.58, respectively.

Also, we analyzed the association of Classifier of Homologous Recombination Deficiency (CHORD) which predicts the status of HRD by utilizing specific single nucleotide variants (SNV), short insertions/deletions (indel) and structural variants (SV) types [9], scarHRD, and expHRD with 23 samples in the TCGA-OV cohort. CHORD exhibited significant associations not only with scarHRD (Spearman’s rho = 0.660; *p* = 6.16e−4) but also with expHRD (Spearman’s rho = 0.484; *p* = 1.63e−2; Fig. S5). Furthermore, we analyzed the distribution patterns of expHRD and CHORD based on mutations and methylation within *BRCA1/2* genes (Fig. S6). Both the Australian Ovarian Cancer Study (AOCS) and TCGA-OV/GDC-OV cohorts showed higher expHRD scores in samples with *BRCA* mutations and methylation compared to the wild type, and CHORD exhibited a similar pattern in AOCS (Fig. S6a, c) and TCGA-OV/GDC-OV (Fig. S6b, d) dataset.

To compare the performance of expHRD with another HRD-associated gene set (n = 230) identified by Peng et al., we performed HRD scoring based on ssGSEA using the HRD-associated genes presented in the paper and tested its ability to distinguish scarHRD high and low [25]. The results showed that our expHRD algorithm yielded notably enhanced prediction potency, as reflected in elevated accuracy and AUC values in both TCGA-OV test set and GDC cohorts (Table 2).

**Table 2:** Performance comparison with other HRD prediction tools on the TCGA-OV and GDC data sets

	Cohort	Sensitivity	Specificity	Accuracy	Precision	F-score	AUC-ROC	AUC-PR	MCC
expHRD	TCGA	0.7105	0.95	0.7931	0.9643	0.8182	0.7988	0.33	0.6283
Guang Peng et al.		0.4737	0.85	0.6034	0.8571	0.6102	0.6583	0.272	0.3201
expHRD	GDC	0.9054	0.5526	0.7857	0.7976	0.8481	0.7738	0.6099	0.5008
Guang Peng et al.		0.5405	0.6579	0.5804	0.7547	0.6299	0.5892	0.4087	0.1882

Subsequent scrutiny of the clinical relevance of expHRD encompassed intra-cohort (TCGA-OV test set, n = 58, Fig. 3c, d) and extra-cohort (GDC ovarian cancer, n = 112, Fig. S5c, d) samples. Recent studies have reported that the HRD score is a clinically proven indicator for ovarian cancer prognosis [21, 30]. As anticipated, patients with scarHRD-high tumours exhibited significantly improved overall survival (TCGA-OV median survival: 1538 days ± confidence interval (CI) 95% (range: 1187–4624), GDC median survival: 1511 days ± CI 95% (range: 1355–2154)) relative to those with scarHRD-low tumours (TCGA-OV median survival: 679 days ± CI 95% (range: 455–1448), GDC median survival: 871 days ± CI 95% (range: 681–1662)), as evidenced in both the TCGA-OV test set (*p* = 0.0004, Fig. 3c) and GDC ovarian cancer cohort (*p* = 0.00037, Fig. S7a). Moreover, patients with high expHRD tumours (TCGA-OV median survival: 2717 days ± CI 95% (range: 1249–4624), GDC median survival: 1442 days ± CI 95% (range: 1264 ~ 1933)) experienced significantly superior overall survival compared to cases with low expHRD (TCGA-OV median survival: 760 days ± CI 95% (range: 627–1448), GDC median survival: 949 days ± CI 95% (range: 690–1946) in both the TCGA-OV test set (*p* = 0.00047, Fig. 3d) and GDC ovarian cancer cohort (*p* = 0.011, Fig. S7b).

We next validated whether the expHRD scoring system could reflect the functional restoration of HRD in recurrent samples with BRCA reversion mutations compared to primary tumors. To this end, we analyzed expHRD in five pairs of primary and recurrent samples with identified BRCA reversion mutations from the AOCS cohort [38]. The analysis revealed that two pairs of samples (AOCS_091 and AOCS_167) exhibited decreased expHRD scores in recurrent tumors, while this pattern was not observed in the remaining three pairs (Fig. S8). These results suggest that the expHRD scoring system, designed primarily to predict scarHRD scores, may have limitations in detecting functional restoration, similar to the scarHRD system.

### Web server development for expHRD calculation

**Fig. 4:**
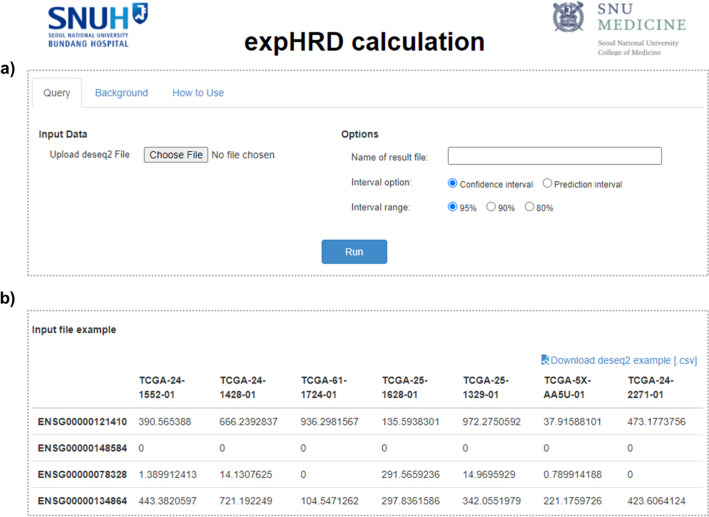
The web interface for expHRD calculation. The front page of the web interface for expHRD calculation demonstrates the query for uploading (**a**) and the input file overview (**b**) of the user’s gene-expression data

**Fig. 5:**
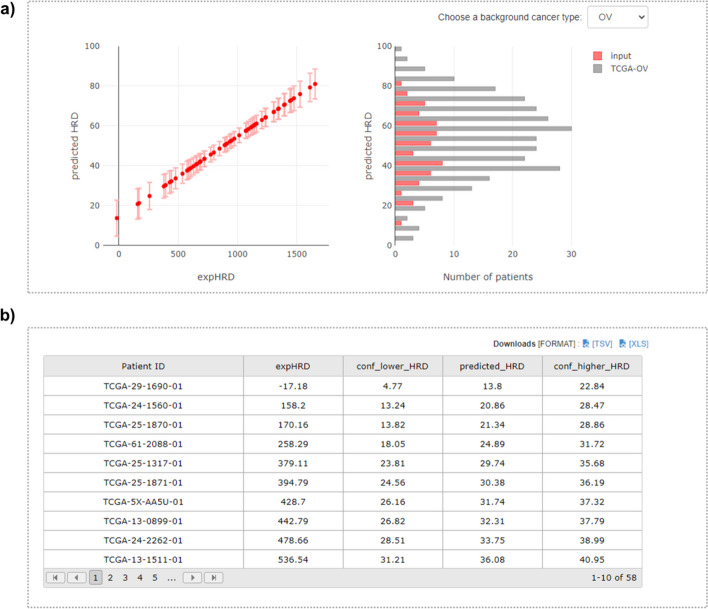
The result page of the web interface. **a** The result page of the web interface exhibited upon selecting “Run” after file upload presents the HRD score graph with the TCGA-OV test sample’s regression function (a, left). The histogram illustrates the distribution of HRD scores (a, right). **b** The result table showcases the calculated expHRD alongside the predicted HRD (p_hrd), both derived from the expHRD outcomes. The values for lower_hrd and higher_hrd are determined by the 95% confidence intervals (CI) of the predicted HRD

To facilitate expHRD calculation, we developed a user-friendly web service enabling researchers to obtain predicted HRD scores akin to scarHRD by uploading their transcriptome data, even for a single tumour. The expHRD webserver (http://www.genome-intelligence-lab.org/expHRD/) is powered by Apache and constructed using the Django framework. Data management is governed by sqlite3 (https://www.sqlite.org/). On the client side, HTML5 (https://html.com/html5/) and JavaScript (https://www.javascript.com/) were employed to create interactive user interface components. Ensuring a responsive user experience, the web server employs bootstrap (https://getbootstrap.com/), jqWidgets (https://www.jqwidgets.com), and plotly libraries (https://plotly.com/javascript/). Noteworthy, expHRD prioritises user privacy, refraining from cookies or personal information collection. Moreover, it guarantees compatibility across major web browsers—Microsoft Edge, Google Chrome, Apple Safari, and Mozilla Firefox. The website is accessible without login requisites and is open to all users. The web server showcases calculated HRD scores (expHRD) upon clicking “Upload” to input DESeq2-based gene-expression profiles of single or multiple samples, followed by the “Run” button (Fig. 4a, b). Users are provided estimated values, along with 95% confidential intervals, derived from expHRD, allowing distribution-based comparative analyses with TCGA-OV samples (Fig. 5a, b). The predicted HRD is a score recalculated from the ssGSEA-based expHRD score of the designated samples and parameters obtained from the linear regression model of scarHRD (Fig. 5).

## Conclusions

In this study, we successfully devised an algorithm, named expHRD, for predicting homologous recombination deficiency (HRD) using transcriptome data. Complementing this, we have established a user-friendly web service, enabling researchers to obtain predicted HRD scores adjusted by expHRD for their own samples. The prediction model was rigorously developed through the application of an elastic net regression within the TCGA-pan cancer training set. Employing the bootstrap technique, we curated the HRD gene set, integral to the expHRD calculation, through single-sample gene set enrichment analysis (ssGSEA). Notably, we observed a robust PCC of 0.768 and 0.633 between expHRD and scarHRD in the TCGA-OV test set and GDC cohort, respectively. Furthermore, expHRD exhibited superior predictive performance for identifying scarHRD-high samples, surpassing a previously established RNA-based HRD prediction methodology [39]. Impressively, expHRD exhibited clinical significance by effectively discerning the overall survival differences among ovarian cancer patients in both the TCGA-OV test and GDC cohorts.

The expHRD technique offers a tailored and clinically relevant RNA-seq-based approach for predicting HRD in tumour-only samples. Within clinical contexts, expHRD holds the potential to furnish valuable insights for anticipating responses to platinum or PARP inhibitor therapies through its provision of predicted HRD scores. Our web service for expHRD calculation represents an essential resource, enabling users to derive expHRD and predicted HRD scores in cases without matched germline data, including cryopreserved tissues, cell lines, or organoids, thereby ensuring a cohort-independent application. Looking ahead, the development of a targeted transcriptomic panel for expHRD computation holds promise as a cost-effective strategy, facilitating seamless integration of our platform within clinical practice.

### Supplementary Information

Additional file1.

Additional file 2.

## Data Availability

The TCGA Pan-cancer SNP Array-Based data, mutation status, and clinical information are available in the GDC data portal (https://portal.gdc.cancer.gov/). TCGA Pan-cancer gene expression and DNA methylation are available at the UCSC Xena browser (https://xena.ucsc.edu/). AOCS data set is available at GEO (Gene Expression Omnibus) with accession number (GSE209964).expHRD web service is available at http://genome-intelligence-lab.org/expHRD/. The source code can be accessible through https://github.com/kimdh4962/expHRD/tree/main
