# Supplementary material for: expHRD: an individualized, transcriptome-based prediction model for homologous recombination deficiency assessment in cancer
Source: BMC Bioinformatics. 2024 Jul 12;25:236. doi: 10.1186/s12859-024-05854-y (PMC11241885; doi:10.1186/s12859-024-05854-y)
Supplement: Supplementary file 1 — Additional file1. [file 12859_2024_5854_MOESM1_ESM.docx]

**Supplementary document**

expHRD: An Individualized, Transcriptome-based Prediction Model for Homologous Recombination Deficiency Assessment in Cancer

Jae Jun Lee, Hyun Ju Kang, and Donghyo Kim *et al*

**Supplementary Methods**

**Gene set profiling**

Enrichr was also investigated to study gene set profile (https://maayanlab.cloud/Enrichr/enrich) [PMID: 23586463]. Enrichr was developed to annotate gene lists by referring multiple gene set libraries including KEGG pathway, ChEA (transcription factor enrichment), Clinvar, and Gene Ontology, counting for 225 libraries. In the profiling analysis, Ontologies filed presenting Biological Process (BP) was considered.

**CHORD calculation**

CHORD (Classifier of Homologous Recombination Deficiency) predicts the status of homologous recombination deficiency by utilizing specific single nucleotide variants (SNV), short insertions/deletions (indel) and structural variants (SV) types [1]. The R package “CHORD” was introduced with default parameter and reference human genome (GRch38), but all variants are filtered in by PASS in variant calling format (VCF). In case of SV, CHORD was designed to only consider specific SV callers (e.g., grids or manta). Other SV callers, for example, BRASS was called WGS data in TCGA, were needed to manual modification of data frame by taking into account SV type and SV length. SV types consist of four types (INV, DEL, TRA, DUP; inversion, deletion, translocation, duplication) in TCGA WGS samples. SV length represents the size of structural variants excluding translocation (TRA) which only denotes the event of translocation.

**AOCS data acquisition**

Australian Ovarian Cancer Study (AOCS) data comprising 126 tumours across 3 primary sites of ovarian cancers (Ovary: 101, Fallopian tube: 3, Peritoneum: 22). The gene expression data was obtained from Gene Expression Omnibus (GSE209964). Clinical information including CHORD score and mutation signatures were obtained from [2].

**Supplementary Figures and legends**


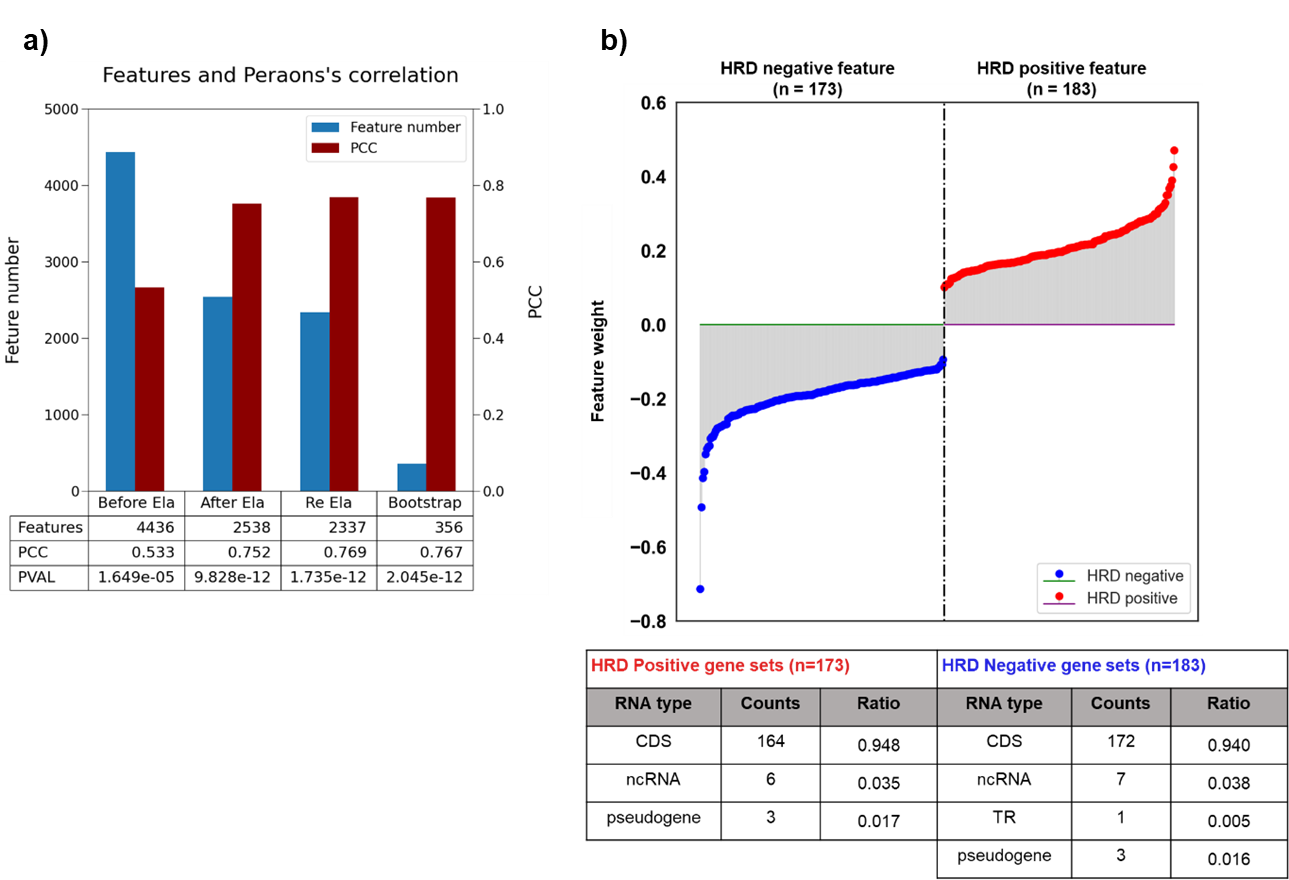


**Fig. S1. Bootstrap to enrich the HRD-related geneset for expHRD calculation**

a) The number of gene features (blue) and PCC (dark red) after indicated machine learning training was demonstrated by a bar plot.

b) Feature weight of selected genes (n = 356) by bootstrap. The selected gene sets, HRD-positive and HRD-negative were additionally analyzed to discern the biological rules of each gene. HUGO Gene Nomenclature Committee (HGNC) biomart provide the information about the gene symbol, ENSG ID, and its biological function. We queried HRD related gene set and classified the gene sets with three RNA type (CDS, ncRNA, pseudogene) and four RNA types (CDS, ncRNA, transfer RNA (TR). Pseudogene) in HRD-positive gene set and HRD negative gene set, respectively. The y-axis represents the feature weight obtained after training. The positive and negative weight indicates the genes showing positive and negative correlation with scarHRD, respectively.

**
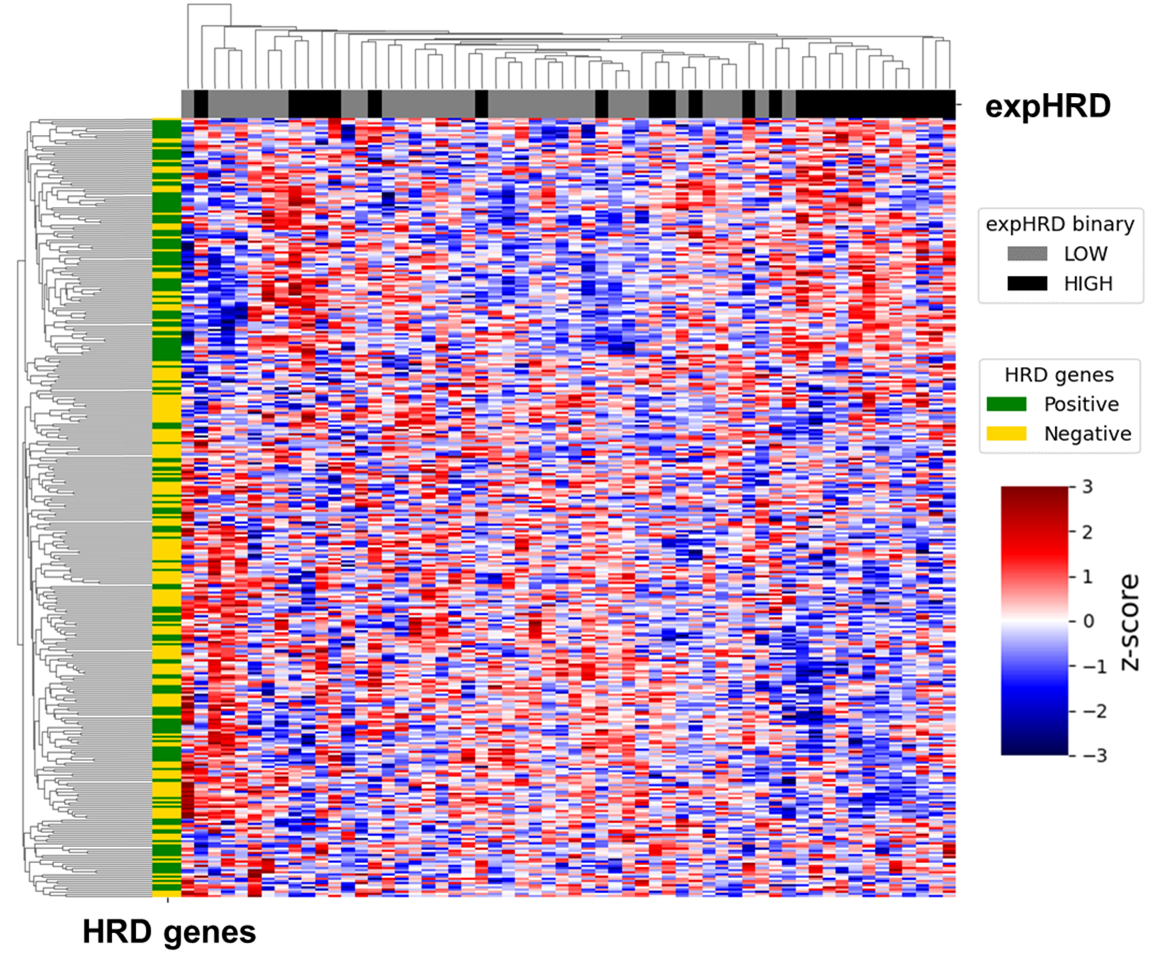
**

**Fig. S2. Gene expression of expHRD signature gene set in TCGA-OV test set.**

TCGA-OV test set (n=58) were plotted based on HRD genes expression value which was normalized by Deseq2 normalization step and standardization with z-score, ranged from -3 to 3. Both column (samples), row(HRD genes) were clustered by hierarchical clustering. Samples were classified into expHRD binary (gray: expHRD low; black: expHRD high). HRD genes were finally obtained after bootstrap process, representing the relatedness to expHRD (green: positive; yellow: negative


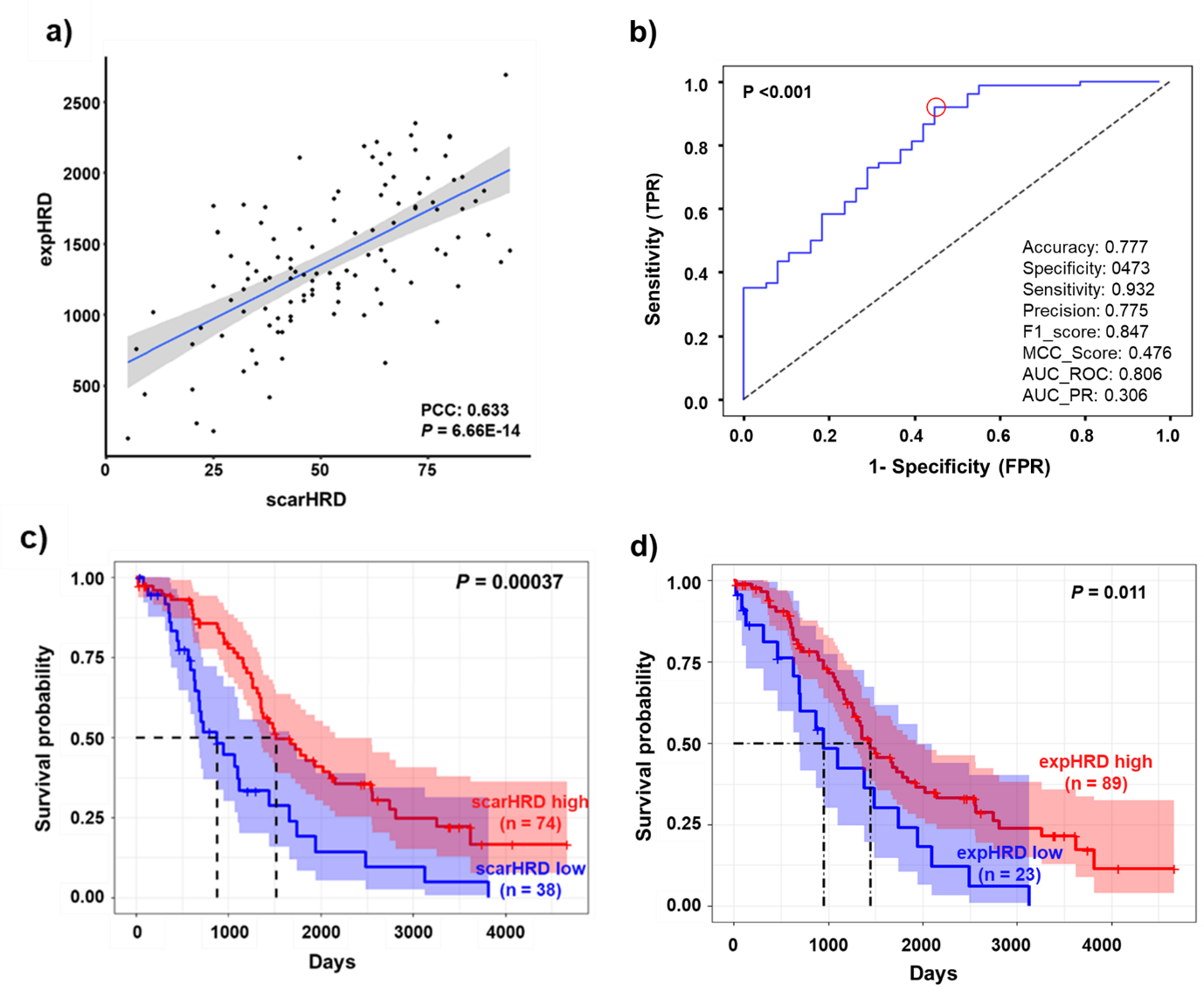


**Fig. S3. Correlation between expHRD and scarHRD in GDC ovarian cancer cohort.**

a) Pearson’s correlation between expHRD and scarHRD in GDC ovarian cancers (n = 112). The blue-colored line and the shaded area represent the regression line and 95% confidence interval (CI), respectively.

b) Receiver operating characteristic (ROC) curve plotted for the sensitivity versus 1-specificty values for predicting scarHRD-high cases using expHRD score in GDC ovarian cancers.


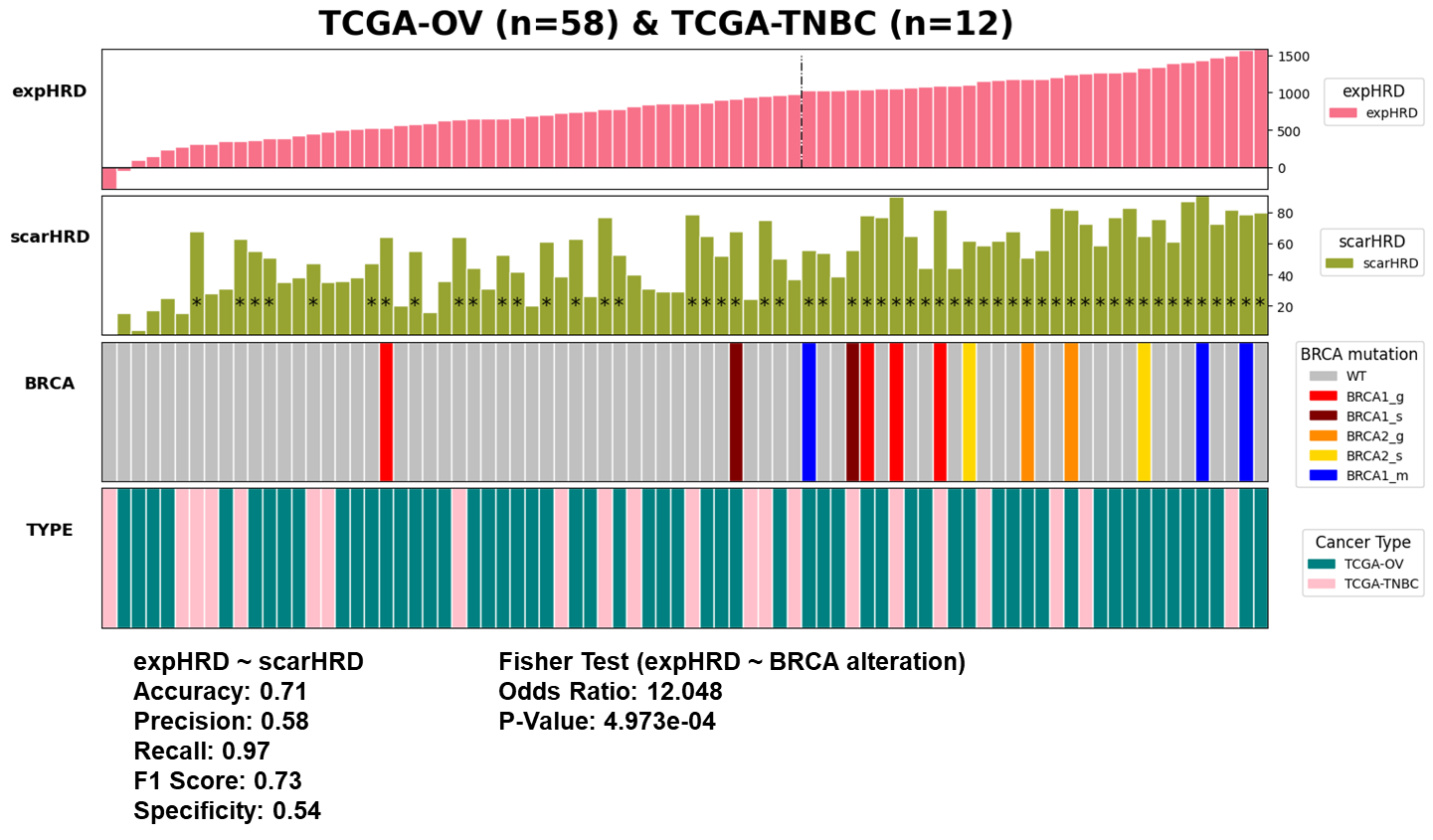


**Fig. S4. Distribution of variants and promoter methylation within BRCA1/2 genes according to the expHRD score in TCGA-OV test and -TNBC cohort**

TCGA-OV test set (n=58) and -TNBC was plotted based on the expHRD score. Somatic or germline variants and promoter methylation in *BRCA1/2* genes were displayed. The dotted line indicates the score 1,000 of the expHRD values. The statistical significance was obtained by Fisher's exact test.


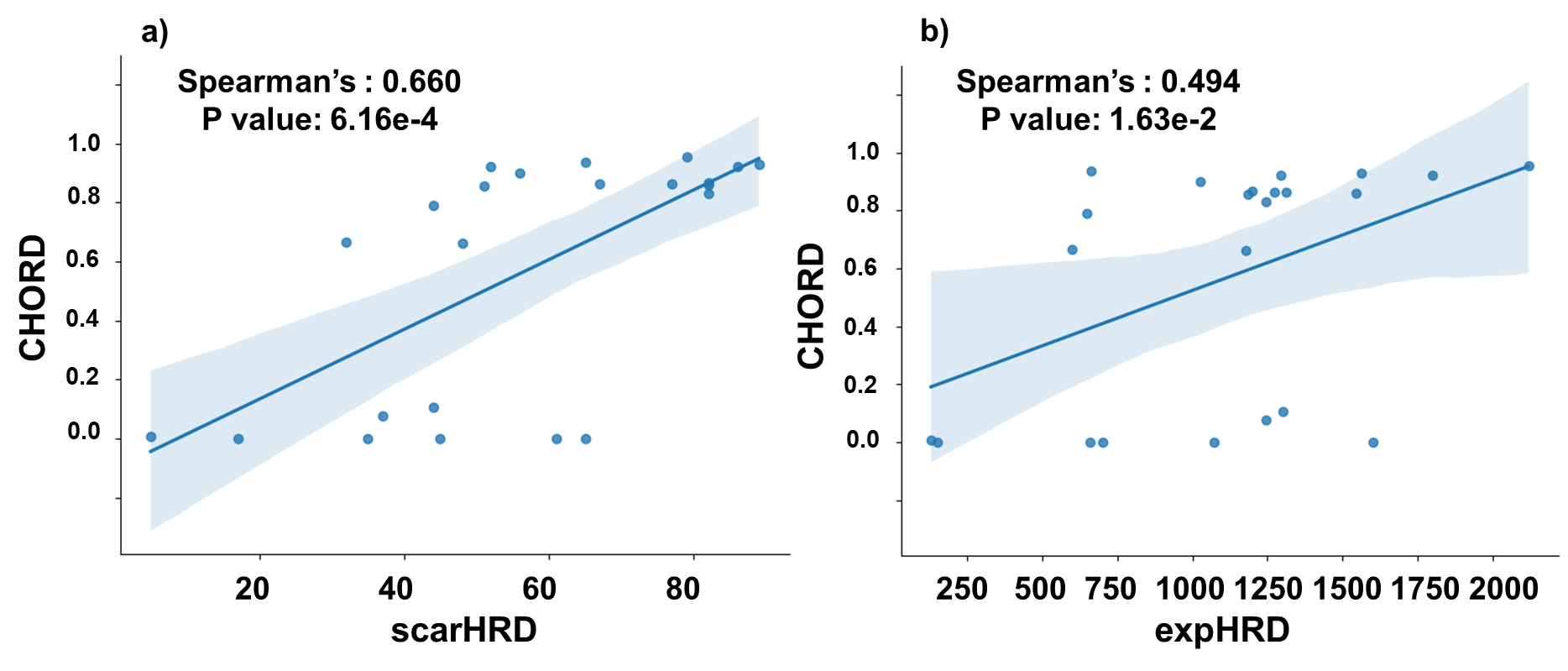


**Fig. S5. Correlation with among CHORD, scarHRD and expHRD scores in TCGA-OV and GDC-OV.**

Total 23 samples analyzed by Spearman’s correlation with a) CHORD and scarHRD, b) CHORD and expHRD, respectively. Shaded area in the plot means Confidence interval (CI =95%) of correlation line.


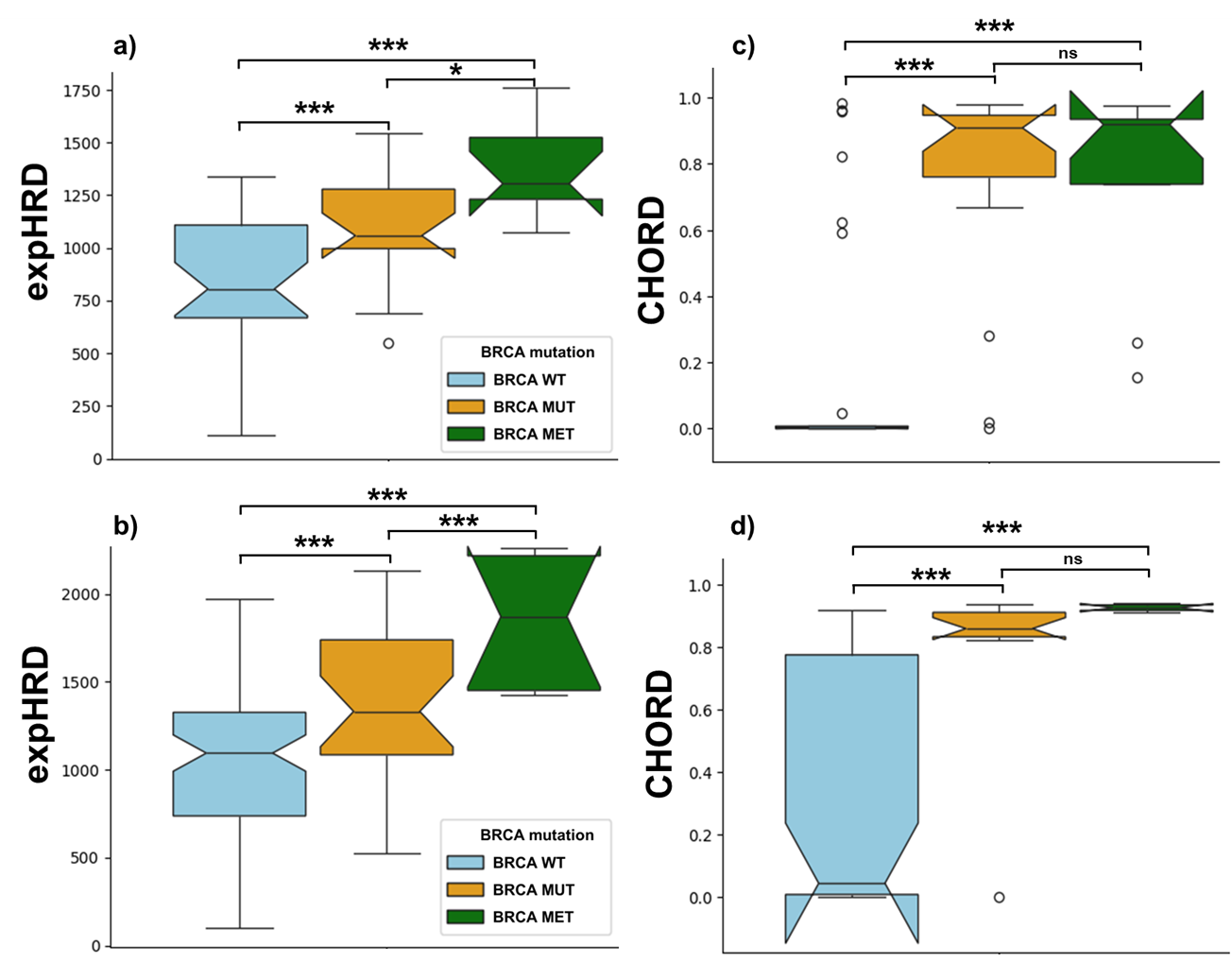


**Fig. S6. expHRD and CHORD analysis by BRCA mutation status**

Comparison of expHRD (a) and CHORD (c) in AOCS. The number of samples in AOCS is 47 (*BRCA* WT: 30, MUT:17, MET: 9). b) Comparison of expHRD (b) in TCGA-OV and GDC-OV. The number of samples is 115 (*BRCA* WT: 80, MUT: 26, MET: 9). d) Comparison of CHORD in TCGA-OV and GDC-OV. The number of samples is 55 (BRCA WT: 39, MUT: 12, MET: 4). Star mark represent the significance of p-value calculated by student’s T-test (ns: non-significant, *: <0.05, **: <0.01, ***:< 0.005, respectively).


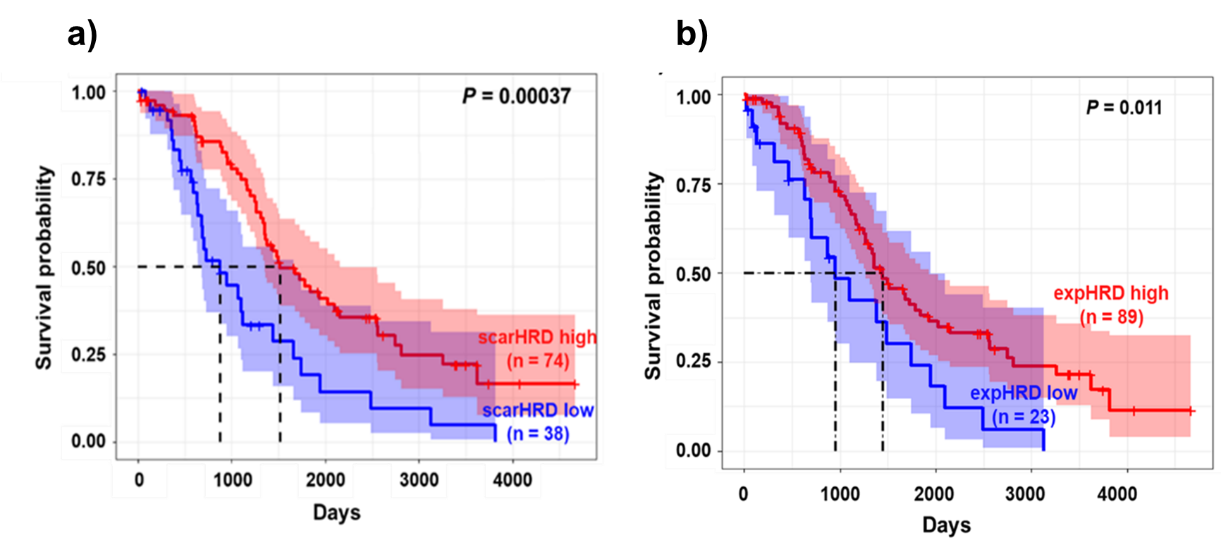


**Fig. S7. Validation of expHRD performance in GDC ovarian cancer cohort.**

Kaplan-Meier overall survival analysis of patients with high vs. low scarHRD (1) or expHRD (b) status in GDC-OV cohort (n = 112). P values were obtained by log-rank test.


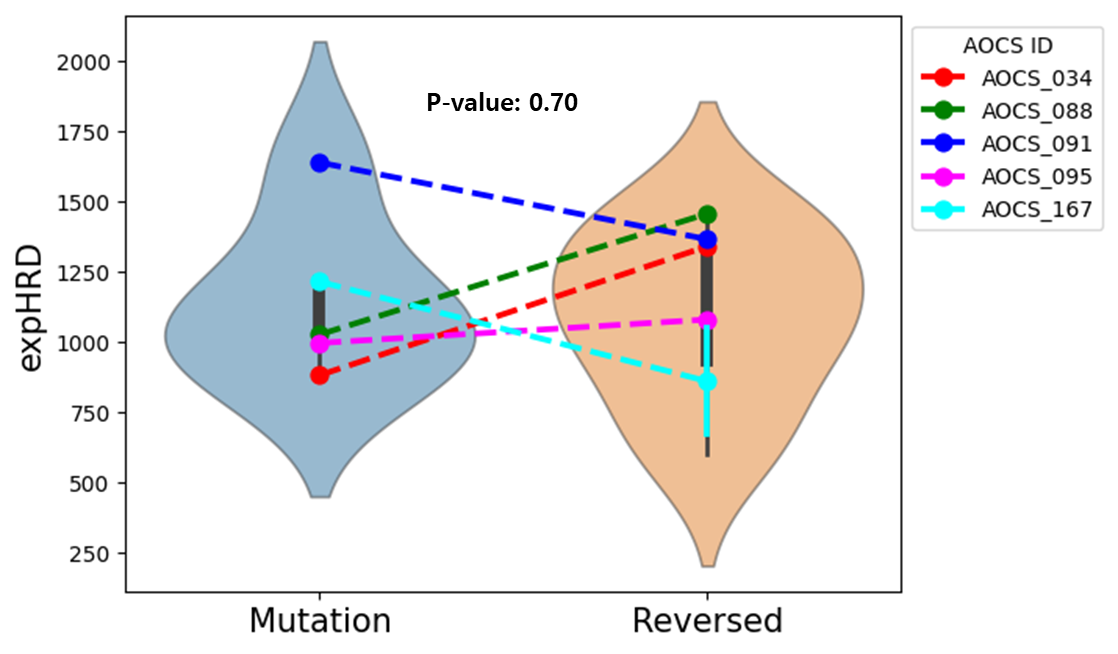


**Fig. S8. expHRD score distribution in BRCA primary and BRCA reversed samples in AOCS.**

Comparisons of expHRD score in BRCA genes (BRCA1, BRCA2) mutation and BRCA reversed in AOCS (total 5 samples). Each dotted line in the plot corresponds to mutation and reversed samples. In the case of AOCS_167, consisting of three reversed samples were included and point of line represent mean expHRD score. P-value calculated by paired student’s T-test with mean expHRD in Mutation and Reversed sample groups

**References**

1. Nguyen L, J WMM, Van Hoeck A, Cuppen E: **Pan-cancer landscape of homologous recombination deficiency**. *Nat Commun* 2020, **11**(1):5584.

2. Garsed DW, Pandey A, Fereday S, Kennedy CJ, Takahashi K, Alsop K, Hamilton PT, Hendley J, Chiew YE, Traficante N *et al*: **The genomic and immune landscape of long-term survivors of high-grade serous ovarian cancer**. *Nat Genet* 2022, **54**(12):1853-1864.
